# Which way in? The Necessity of Multiple Approaches to Transcatheter Valve Therapy

**DOI:** 10.2174/1573403X09666131202123326

**Published:** 2013-11

**Authors:** S. Bleiziffer, M. Krane, M.A. Deutsch, Y. Elhmidi, N. Piazza, B. Voss, R. Lange

**Affiliations:** Clinic for Cardiovascular Surgery, German Heart Center Munich, Munich, Germany

**Keywords:** Transcatheter valve implantation, transapical, transfemoral, subclavian, transaortic.

## Abstract

TAVI (transcatheter aortic valve implantation) is a less invasive treatment of the stenotic aortic valve while avoiding midline sternotomy and cardiopulmonary bypass. A crimped biological valve on a self-expanding or balloon-expandable stent is inserted antegradely or retrogradely under fluoroscopy, and deployed on the beating heart. Among the worldwide TAVI programs, many different concepts have been established for the choice of the access site. Whether retrograde or antegrade TAVI should be considered the superior approach is matter of an ongoing debate. The published literature demonstrates safety of all techniques if performed within a dedicated multidisciplinary team. Since there is no data providing evidence if one approach is superior to another, we conclude that an individualized patient-centered decision making process is most beneficial, taking advantage of the complementarity of the different access options. The aim of this article is to give an overview of the current practice of access techniques for transcatheter based valve treatment and to outline the respective special characteristics.

## INTRODUCTION

**Fig. (1):**
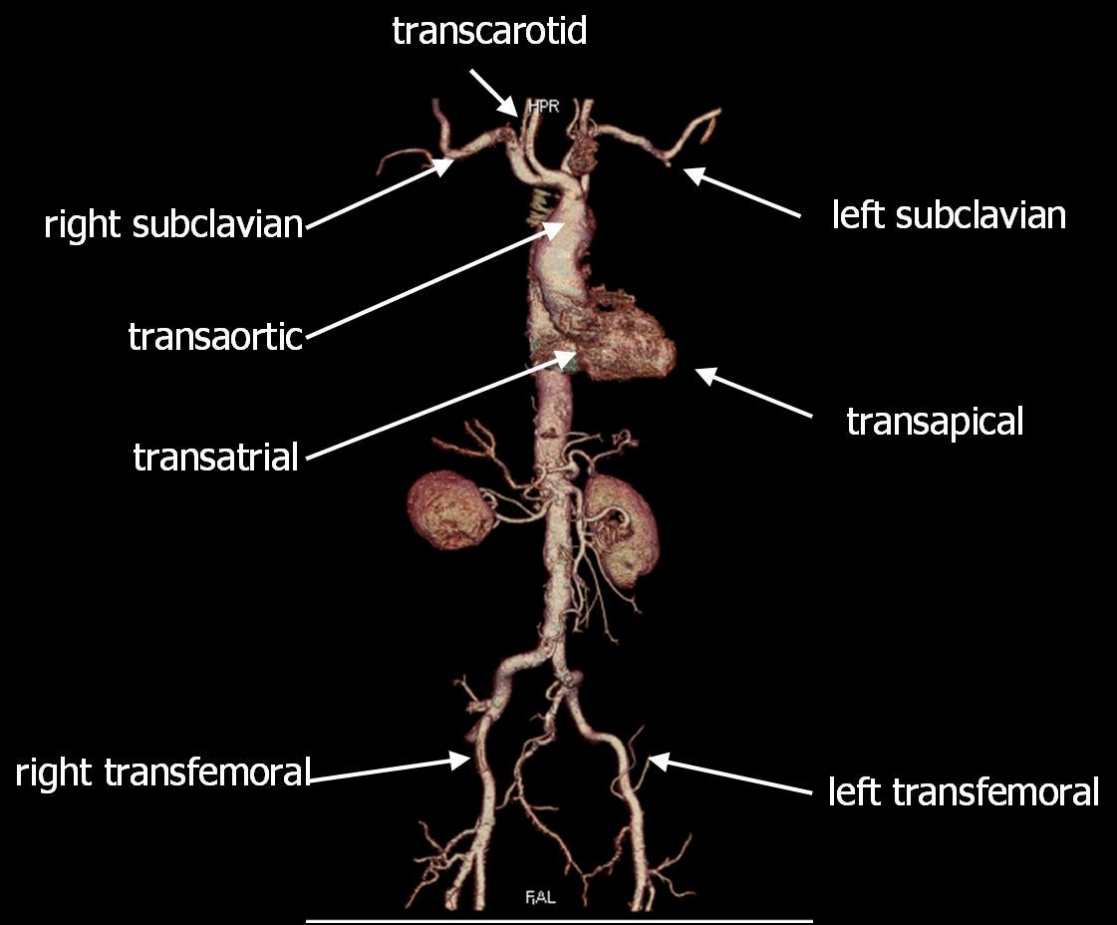
CT scan of the thoracic and abdominal aorta, demonstrating the various access options for transcatheter valve procedures.

Whether retrograde or antegrade TAVI should be considered the superior approach is matter of an ongoing debate. “TAVI” (transcatheter aortic valve implantation), or recently often termed “TAVR” (transcatheter aortic valve replacement) refers to a less invasive treatment of the stenosed aortic valve. A crimped biological valve on a self-expanding or balloon-expandable stent is inserted antegradely or retrogradely under fluoroscopy and deployed on the beating heart. Antegrade access to the aortic valve is achieved through the apex of the left ventricle which requires a left anterolateral minithoracotomy. Retrograde, transarterial access is usually achieved transfemorally, or less frequently through the subclavian or carotid artery, or the ascending aorta (see Fig. **1**). While transfemoral TAVI can be achieved percutaneously, the subclavian and transcarotid access are mostly performed by means of a surgical cut-down, while the transaortic access requires (partial or full) sternotomy or right lateral thoracotomy. 

Newer transcatheter based valve treatments are increasingly gaining interest, such as transcatheter mitral valve replacement, valve-in-ring implantations, valve-in-valve implantations in aortic, mitral, or tricuspid position. There is a variety of access options for these techniques.

The aim of this article is to give an overview of current practice of access techniques for transcatheter based valve treatment and to elucidate the respective special characteristics. We conclude that an individualized patient-centered decision making process is most beneficial, taking advantage of the complementarity of the different access options. 

## ANTEGRADE TRANSSEPTAL ACCESS

The first reported human transcatheter aortic valve implantation has been performed by Alain Cribier in 2002 via a antegrade transseptal access [1]. A 24F sheath was inserted into the right femoral vein, and the interatrial septum was balloon-dilated. The percutaneous heart valve was advanced through the sheath, across the interatrial septum and the mitral valve, and was positioned within the diseased stenotic aortic valve. Because of the complexity of this approach, it was abandoned after the introduction of the transfemoral and transapical routes. Today, the transseptal route has a significant value to access the mitral valve [2]. 

## TRANSFEMORAL 

During the first attempts of retrograde transfemoral valve implantations in 2005 [3, 4], the implanters had to deal with larger sheaths of 24-25F, which exclude many patients for this approach and bear a high risk of vascular access complications. Nowadays, device profiles are reduced to 16-18F for the Medtronic CoreValve and Edwards Sapien prostheses, which were both CE marked already in 2007. The retrograde transfemoral access is considered first choice in many TAVI centers because of its obvious minimal invasiveness. With several ten thousands transfemoral TAVIs having been performed worldwide, a broad experience has been gained during the last years. Still, vascular complications at the femoral access site are reported in 10-20% in large patient series [5, 6] being described to be associated with increased mortality [7]. Potential vascular access site complications include vessel rupture, dissection of the iliac artery or the aorta, vessel occlusion, bleeding and hematoma, or false aneurysm with potential need for catheter or surgical vascular intervention and transfusion. Therefore, meticulous patient selection for the retrograde transfemoral access is paramount. The access vessel diameter should be at least 6mm, without significant turtuosity, stenosis, or calcifications. Previous surgery or stent implantations in the aorta or iliac and femoral arteries should be considered contraindications for transfemoral TAVI. It can also be recommended to perform a surgical cut-down of the femoral artery if there is doubt about the efficacy of percutaneous closure devices, e.g. if calcifications are present at the puncture site. Newer sheath generations with smaller size and new features such as expandability may increase safety and broaden indications also in difficult anatomies [8, 9]. Finally, transfemoral TAVI can easily be performed without general anaesthesia, however, no consensus is yet made if sedation is advantageous over general anaesthesia [10, 11].

## SUBCLAVIAN

The second transarterial retrograde access route for TAVI is the subclavian artery access. The first case reports on this new approach were published in 2008 [12, 13]. At the author’s institution, approximately 6% of all TAVI procedures are performed via the subclavian artery. The right subclavian artery [13] is rarely used, due to an unfavourable implantation angle. The vast majority of subclavian TAVI cases are performed using the CoreValve prosthesis, because of a small introducer sheath. In addition, for Sapien implantation, a straight portion of a certain length of the artery is needed to place the crimped valve onto the balloon, which leads to a more complex subclavian procedure. Only few cases of Sapien implantations through the subclavian artery have been described [14, 15]. Usually, a surgical cut-down is performed to access the subclavian artery and to introduce the sheath after placement of purse-string sutures. More recently, a percutaneous access technique has been described [16]. Although general anaesthesia might be beneficial in terms of analgesia, a certain proportion of subclavian TAVI cases are performed in sedation [17, 18]. There are several distinctive conditions to be considered for the subclavian TAVI procedure: a patent LITA graft may be at risk with an occlusive sheath in the subclavian artery. However, there is a body of case reports demonstrating that subclavian TAVI can be performed safely in patients with a patent LITA graft [19-21] if the artery is large enough, or if the sheath is not advanced across the LITA origin. A previously implanted permanent pacemaker at the access site is a relative contraindication for subclavian TAVI to avoid the risk of wire injury. As the subclavian artery wall is thinner and more frail than the femoral artery, very careful handling is required to avoid vessel complications such as dissection. Furthermore, the anatomical proximity to the brachial nerval plexus requires special attention to avoid neurological complications [22]. A propensity matched study of the Italian TAVI registry demonstrated comparable procedural and 2-years results of subclavian and transfemoral TAVI [23] in 141 patients per group. It was therefore concluded, that the subclavian access “should be considered a valid option not only when the femoral approach is impossible but also when it is difficult, albeit feasible” [23].

## TRANSAORTIC

The direct transaortic route for TAVI was first reported in 2009 as a bail-out strategy in a patient with unfeasible transfemoral, subclavian or transapical access [24]. To access the ascending aorta, a full or partial upper sternotomy, or a right anterolateral thoracotomy can be performed through skin incisions of 6-7cm. The placement of purse-string sutures, usually in the right lateral upper quadrant of the ascending aorta, is required to obtain safe hemostasis. In fact, cardiac surgeons are familiar with suturing and cannulating the aorta for all conventional procedures with heart-lung-machine. As a porcelain aorta is considered a contraindication for the transaortic approach, careful CT assessment of the calcium distribution in the ascending aorta is required [25]. Today, there is little literature on results after transaortic TAVI in larger series. Transaortic CoreValve implantations with the 18F Cook sheath and the transfemoral delivery catheter have successfully been performed through partial sternotomy or lateral thoracotomy in up to 25 patients [26, 27]. Transaortic Sapien implantations through upper sternotomy were performed with the transapical sheath and the valve crimped in the inverted direction for retrograde implantation [28]. With this method, placement difficulties with the Sapien valve during transaortic implantation due to design features of the introducer sheath have been experienced [29]. These difficulties are already addressed by the next generations of Sapien delivery device (Ascendra plus). While the transaortic route is still less frequently used in most centers, few implanters prefer this route to transapical TAVI to avoid myocardial injury at all. By all means the transaortic route is a good option for patients who cannot have a transfemoral or subclavian TAVI, and the transapical route is unfavourable, e.g. because of severely impaired left ventricular function or previous left thoracotomies. Longer-term results in larger series are lacking.

## TRANSAPICAL

The first human transapical aortic valve implantation has been performed by the group of John Webb with the antecessor valve of the Sapien prosthesis in 2005 [30]. Initially, there have also been attempts to implant the CoreValve prosthesis transapically [31] within the context of a feasibility study, but the device is no longer available. Advantages of the transapical procedure are a short distance from the sheath to the annulus and the antegrade implantation route facilitating exact positioning, the possibility to accommodate larger sheaths up to 36F, and virtually no access limitation as the apex can be exposed in almost every patient. Thoracic deformations precluding a lateral incision, which are very rare, and a very poor left ventricular ejection fraction may be seen as contraindications for transapical TAVI. The transapical technique is well standardized today and used at most centers as the second option for TAVI patients if a transfemoral implantation is not feasible. The left thoracotomy incision can be performed truly less invasive through a 6cm skin incision. Haemostatic control of the apex is the critical step during the procedure. Several surgical techniques have been described for apical management. At the author’s institution, the pericardium is opened if at all possible even in redo patients to ensure the localization of the LAD [32]. Puncturing the real apex can reduce the tension on the suture [33], while most centers prefer to puncture slightly lateral and above the real apex, where the tissue exhibits more strength and the myocardium is thicker [32, 34]. In any case, epicardial fat should be avoided. The use of 2-0 or 3-0 Prolene purse-string or mattress sutures with 4-8 Teflon pledgets and deep transmural or non-penetrating bites have been described [32, 33, 35]. Ventricular puncture should be performed centrally within the sutures [33]. During sheath removal and tightening of the purse-string sutures, rapid ventricular pacing can be installed to lower the systolic blood pressure [32]. Large multi-center data have demonstrated reliable results after transapical implantation of the Edwards Sapien valve [6, 36]. Rahnavardi and coworkers conclude from a large systematic review of publications on transapical procedures that the results suggest transapical TAVI as having less vascular complications, decreased use of contrast or fluoroscopy and possible different adverse neurologic outcomes [37]. Second generation transcatheter heart valves have recently been CE marked for transapical implantation, such as the Symetis Acurate [34], the JenaValve [38], and the Medtronic Engager [52] prosthesis. The next step towards minimizing trauma and apical bleeding is the development of apical closure devices for safe hemostasis and potential percutaneous apical access [39]. A couple of devices are under investigation and data from first-in-human implantations are expected shortly. 

## TRANSCAROTID

Another alternative retrograde route for TAVI implantation was recently introduced by Modine and coworkers [40]. The left carotid artery was exposed, and a CoreValve prosthesis was implanted through an 18F sheath under cerebral oxymetry in twelve patients. Having demonstrated the feasibility and short-term success of this new approach, the authors conclude that the transcarotid access adds yet another tool to the armamentarium for TAVI procedures.

## TRANSATRIAL /TRANSJUGULAR /FEMORAL VEIN

A novel emerging field in transcatheter valve procedures is valve-in-valve or valve-in-ring implantation in degenerated biological surgical prostheses or failed repair procedures of the mitral and tricuspid valve. Therefore, some other access sites than for the aortic valve are required. Access to a mitral ring or bioprosthesis can, however, been achieved with the well-established transapical technique [41, 42]. There are a couple of case reports describing alternative procedures, such as venous femoral transseptal mitral valve-in-valve implantation [43], or venous transjugular transseptal mitral valve-in-valve implantation [44]. The transjugular access has also been used to access the tricuspid valve [45]. Another alternative is the access through a right lateral thoracotomy and direct atrial access [46, 47], where both atria can be approached. A large registry of 70 mitral valve-in-valve or valve-in-ring procedures revealed the use of the transapical access in 85.7%, of the transseptal access in 10% and of the transatrial access in 4.3% [48]. Longer-term results in larger series have to be awaited.

### Which way in?

Among the worldwide TAVI programs, many different concepts have been established for the choice of the access site. The most current expert consensus report does not give a general recommendation for access site selection [49]. In fact, there is no data providing evidence if one approach is superior to another. Randomized studies have not been performed. Therefore, some argue that a balanced use of transfemoral and transapical procedures should be maintained until appropriate data is acquired [50]. In contrast to that, the most current practice is the “transfemoral first” approach which is build on the degree of invasiveness: from transfemoral (least invasive, first choice) to transapical/transaortic (most invasive) [51]. However, it is to note that not only the length of an incision defines invasiveness. Less desirable, some centers are limited by using only one transcatheter valve type (e.g. the CoreValve can not be implanted transapically).

A randomized comparative study would be desirable in order to draw a final conclusion if there are advantages of one access technique. Unfortunately, a couple of difficulties in the potential design for a randomized trial precluded its realization to date: First, only patients eligible for both transfemoral and transapical TAVI could be enrolled. Second, only the same type of prosthesis should be implanted. Currently, the Edwards Sapien prosthesis is the only valve available for both antegrade and retrograde implantation. On the other hand, this would lead to a prosthesis specific study rather than focusing on the access issue. Through competence splitting in most TAVI centers, transfemoral TAVIs are performed by interventionalists whereas transapical TAVIs are performed by surgeons, adding another bias.

**Fig. (2):**
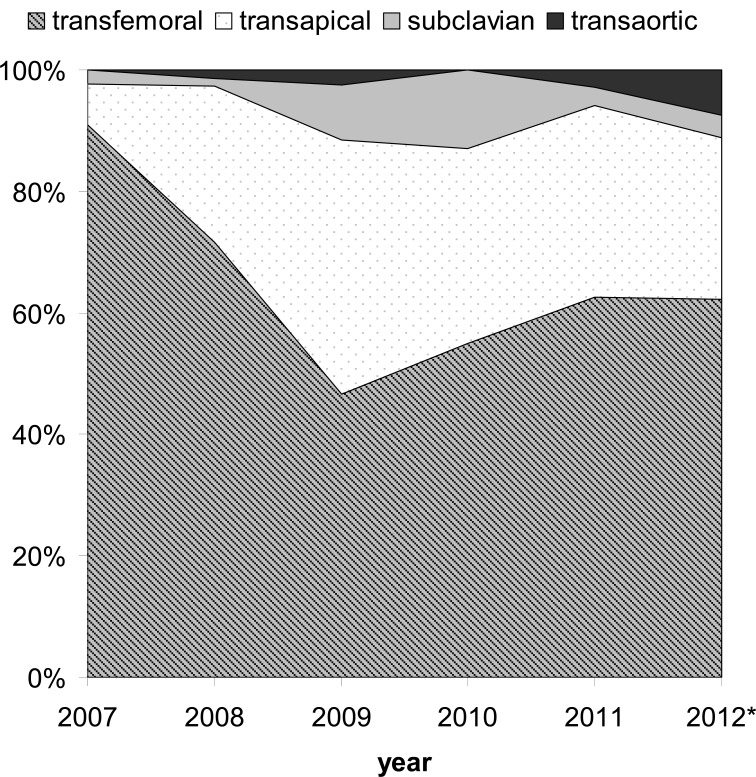
Distribution of access sites from the TAVI program at the German Heart Center Munich from 2007-2012. Absolute implantation numbers were n=44 in 2007, n=148 in 2008, n=165 in 2009, n=162 in 2010, n=171 in 2011, n=135 in 2012 (* from January to July).

To the authors’ opinion, the available access sites for catheter based valve therapies should not be seen competitive. At the Geman Heart Center Munich, a very individualized patient evaluation is performed based on a “transfemoral first” approach, without forcing the transfemoral access in every patient. The decision making process has been refined over the years, and thus, the proportion of the access sites used has changed over time (Fig. **2**). Our concept is based on a complementary approach using several devices (Medtronic CoreValve, Edwards Sapien, Medtronic Engager, JenaValve) and alternative routes tailored to the anatomy and the comorbidities of the individual patient. All procedures are performed by the same dedicated team of cardiac surgeons and interventional cardiologists trained for all different procedures. For example, a patient with diseased femoral and subclavian arteries and a severely impaired ventricular function would be scheduled for transaortic rather than transapical implantation, or a heavily calcified or bicuspid valve would rather be treated with a self-expandable prosthesis, or a less calcified valve would be treated with a second generation transcatheter valve prosthesis (which is available transapical only to date). 

In summary, a couple of different access options with specific characteristics are available today for transcatheter based valve treatment. The published literature demonstrates safety of all techniques if performed within a dedicated multidisciplinary team. We conclude that an individualized patient-centered decision making process is most beneficial, taking advantage of the complementarity of the different access options.
